# On Dreaming and Somnambulism in Relation to the Functions of Certain Nerve-Centres

**Published:** 1864-07

**Authors:** W. S. Savory


### ON DREAMING AND SOMNAMBULISM IN RELATION TO THE FUNCTIONS OF CERTAIN NERVE-CENTRES.

By W. S. SAVORY, ESQ., F. R. S.

In the first portion of the discourse a sketch was given of the general plan of construction of the nervous system, from the simplest to the more complex forms. Then the different kinds of reflex action were alluded to, and the several centres indicated which, with greater or less certainty, are known to be concerned in their production. Thus the simplest and most universal form of reflex action, called excito-motor, being not necessarily attended by sensation or consciousness, was referred to the spinal cord and medulla oblongata. Sensori-motor actions, those which involve sensation or consciousness, but which do not necessarily arouse ideas, were referred to certain ganglia beyond, such as the optic thalami—the corpora striata being, perhaps, concerned in motion—and the olfactory, optic, auditory, and gustatory ganglia; these, in all probability, collectively constituting what is called the sensorium, each kind of sensation being produced through its own proper ganglion. Lastly, those acts which involve ideas, and which are therefore called ideo-motor, were referred to the cerebrum.

The production of what are called subjective sensations was accounted for by the fact, that if an impression be made on any part of a sensitive nerve, even if in the centre to which it passes, the sensorium perceiving that impression, refers it, not to the part of the nerve which is impressed, but to its periphery, to the part to which the filaments are distributed.

Then sleep was described as a state of rest of the sensorium and cerebral lobes. Its leading phenomena are the result of the suspension of their functions—the suspension of consciousness and of the mental faculties generally. Sleep is to the brain what rest is to every organ of the body.

Of dreaming it was said: When sleep is partial or imperfect the functions of those ganglia whose rest is sleep will not be entirely suspended. They will remain, in some measure, active. Impressions will still be recognised by the cerebral hemispheres, and will give rise to ideas. Thus dreams are produced. Dreams, therefore, are the result of imperfect exercise of the hemispheres when in a state of partial repose. There may be total abstinence of consciousness of external things, and yet withal a state of mental activity varying greatly in degree and duration. Dreams, then, occur when sleep is not profound.—They cannot arise during complete repose.

There is no sufficient reason to deny the existence of a period of complete unconsciousness—of complete suspension of the mental faculties. Doubtless the brain, like other organs, is at times in absolute repose. While dreaming, sleep must be considered imperfect.

Again, if the general view here expressed be the correct one, that dreaming is the natural condition of imperfect repose, there is no good reason for denying the supervension of dreams at any period of sleep.

There can be little doubt that dreams are very transient, but the evidence of the extreme rapidity which has been assigned to all of them is defective. Of course the duration of dreams bears no comparison to that of the events and circumstances which they picture. But looking at the relation of dreaming to somnambulism, their frequent occurrence, the period occupied by day-dreams, and the time that disturbed sleep and other indications of dreaming will sometimes last, it is, perhaps, more reasonable to conclude that some dreams are not so instantaneous as many imagine.

It is highly probable that all animals with cerebral hemispheres dream. This conclusion, which naturally follows on the view here taken of the nature of dreams, is confirmed by observation.

When impressions reach the cerebral hemispheres they arouse ideas. Impressions may be objective, arising from without, what we call real; or subjective, arising from within, what we call fancies. The exciting cause of dreams, then, may come from without or from within.

As the chief feature of sleep is a state of unconsciousness, so, in general, the remarkable feature of dreaming appears to be an absence of the power of the will over the current of thought and over action. Thus ideas are aroused in rapid succession without guidance or correction, but no volitional acts are accomplished. Hence the frequent incoherence and inconsistency of dreams. Of course there are all degrees of this. Dreams may be and, often are, consistent and rational, whether from the more complete exercise of the cerebral lobes, or from the nature of the impressions which excite them; but oftentimes they are characterized by a strange want of regulation and co-ordination of the ideas which represent them. When, therefore, we dream what we call nonsense, it is because there is a partial or complete absence of voluntary control over the current of thought, and because we cannot compare our conceptions and ideas with surrounding objects and circumstances, and thus correct them.

The extent to which the ideas that constitute dreams are coherent, depends probably, in great measure, upon whether they are habitual or strange to the waking state. When the will is not alert, old ideas are more apt to be orderly than new ones, for the former may fall into their accustomed sequence, whereas the latter have not even habit to arrange them. Every one knows, for instance, how an idea excited by an impression may forthwith arouse a train of others which have been before associated with it. Thus, for the most part, dreams are rational in proportion as they arise out of existing circumstances.

In comparing, then, the condition of the mind in dreaming with its active state while awake, we are led to notice these distinguished features:—

The will is in abeyance. It ceases to control, or rather to direct, the current of thought.

The correcting influence of external impressions is suspended.

Impressions conveyed to the brain when awake excite ideas, which are, for the most part, in a healthy mind, subjected to the regulating influence of the will; and if they give rise to acts these are voluntary and rational. But when the influence of the will is suspended, and it ceases to direct the current of thought, the acts which such ideas thus produced may at once excite are often strikingly irrational or altogether absurd.—Such examples may be seen in persons intoxicated by alcohol, or laughing gas.

As in these cases, so, in the same way, it is doubtless possible sometimes to lead the ideas during sleep, when not profound, by by means of external impressions, and even to determine their nature. Thus gloomy ideas may be suggested by a dull tone of the voice, while cheerful ones may be aroused by lively sounds. In the same way the ideas may be adapted to certain conditions in which a person may be placed. Every work on the subject contains some illustrations of this. And just as a dream may be started by an external impression, so an impression made upon any of the senses during a dream will oftentimes fall into the current of ideas then flowing through the mind. Thus a noise may become the report of firearms, or the shout of a multitude, or a peal of bells, or something else, according to the subject of the dream.

The nature of dreams, in their relation to the absence of the correcting influence of surrounding circumstances, is well illustrated by the effect of darkness and silence on delirium. The phantoms which then arise, the correcting influence of external circumstances being shut out, will often at once disappear in the presence of light or at the sound of a well-known voice.

There is a peculiar condition of the mind often occurring in some persons which is well expressed by the term day-dreaming. In this state the ideas are allowed to flow on without control. They are not restrained by any effort of the will. In the worst form they are not even co-ordinated. One idea suggests another, and so on until the thoughts have wandered far away from the original subject. Nothing is seen but the vision of fancy. The most improbable, nay impossible, prospects are conjured up and contemplated either as present or future realities, and no attempt is made to check or control the most extravagant or erroneous conclusions. Here, however, at least at the outset, there is an indisposition rather than an inability to reason. When in this state a person is said to build castles in the air. The mind is wholly withdrawn from the consideration of external and surrounding circumstances, and revels in the luxuriance of its thoughts. This is really a dreaming state, although, owing to the activity of the sensorium, day-dreams are more readily corrected by external circumstances.

Indeed, if we attend at all to the state of our mind, we must observe that it varies widely, and during a considerable portion of our waking hours, in many persons, especially the young, its condition is not far removed from day-dreaming. When we are not at work, when the attention is not fixed, during periods of leisure, the intellect is wont to escape insensibility from the control and direction of the will, and to wander far on into the regions of thought, one idea suggesting another, which forthwith takes its place. One step farther, and the influence of surrounding circumstances is almost shut out, and the imagination revels without restraint. Then we may be said to dream.

Day-dreaming, or reverie, and the dreams of sleep are connected by a peculiar phase of dreaming which sometimes occurs when the sleep is unusually light, or more often when we are awakening out of sleep. In this state the sensorium is more or less active. We are conscious. It is characterized, moreover, by a partial and imperfect control over the current of thought, and a voluntary effort, in some degree successful, is made to prolong agreeable ideas and to dispel gloomy ones. This condition must be familiar to every one as occurring at the dawn of day before rising.

Again, every one knows that a dream out of which we have awakened is very liable to occur if we fall asleep again. Such cases tend to establish the relation between our sleeping and waking thoughts. By watching and analyzing the phenomena we can observe how insensibly they pass into each other, the vision of our sleep, rising into ascendancy as the influence of external impressions and of the will is withdrawn.

Of somnambulism. Now just as dreams may be regarded as due to partial activity of the cerebral lobes, when in a state of imperfect repose, so may the condition termed somnambulism be regarded as essentially the result of a state of more or less complete activity of the sensorium, the hemispheres beyond being nevertheless at rest.

In what may be called the purest form of somnambulism various acts may be accomplished in the most perfect manner, as the direct result of impressions, without any evidence of the intervention of ideas. But although in somnambulism impressions do not necessarily arouse ideas, yet they nevertheless produce sensations and determine sensori-motor acts. That they react on the sensorium, and are not merely excito-motor in their nature, is evident from their results. Sounds are sometimes heard, and objects recognized by the sight and touch.

The term somnambulism appears to be very loosely employed. In its common acceptation a combination of dreaming and somnambulism is implied. Perhaps, indeed, this is the most common form of somnambulism. Not only is the sensorium active, but the hemispheres themselves are partially awake. But although the description usually given of somnambulism would imply an active state of the cerebral hemispheres as well as of the sensorium, would include a state which is essentially that of dreaming —so that somnambulism is described as an "acted dream"— yet, while admitting that this more complicated condition may be a common one, it is in the highest degree important to recognize the fact, that a state may occur in which some or all of the sensorial centres are active, the cerebrum itself being nevertheless in a state of complete repose; the actions which result being simply sensori-motor or instinctive ones. Thus no ideas are aroused, and nothing is remembered. This may be described as the simplest and purest form of somnambulism.

It seems, then, that the clearest and most correct idea will be obtained of the two states, and of their relation to each other, by regarding them as manifestations of various degrees of activity of those centres which in profound sleep pass into a state of complete repose; that either alone may be active, while the other is at rest, or both together in imperfect sleep may exhibit all degrees of partial activity, in various proportion, short of that thorough and complete exercise of their functions when wide awake.

It appears that this view will afford an insight into certain facts which do not otherwise admit of explanation.

Dreaming is more frequent than somnambulism, because of all portions of the nervous system the cerebral lobes are the most sensitive to variations in the quantity and quality of blood circulating through them. Witness the effects of alcohol, ether, and chloroform.

Somnambulism is more common in the young, because then the sensorial centres are more prone to activity. With regard to dreams the rule is less general, being influenced by habits of mental exercise.

The remembrance of dreams, not of somnambulistic acts. It is important to note the absence of memory in somnambulism. It appears that acts purely somnambulistic are never remembered or recalled. This seems to point to their nature. They find their parallel in instinctive actions.

The dexterity and accuracy of somnambulistic act; for example, of muscular movements in walking. Perhaps in this case the cerebellum is awake also.

The somnambulist walks across a narrow plank over a frightful chasm steadily and without fear. Why? Because the act is a sensori-motor one, and no idea is called into play. For the same reason, a person wide awake may accomplish the same feat in the same manner if the danger be concealed from him—if he have no idea of it. In the former case, he may see it, but has no idea of it; in the latter, he has no idea of it because he cannot see it.

In this view of the matter, therefore, the actions of the somnambulist are essentially of the nature of instinctive ones. A careful annalysis of them show that they possess this character. In the simplest and purest form of somnambulism, as in the simplest and purest examples of instinctive acts, there is not any satisfactory evidence of the operation of the intellectual powers. In either case is seen the adaptation of means to ends; but there is no evidence of the intervention of ideas, of the calculation of consequences, of reason. This difference, however, must be noted in the two cases: The operations of instinct are invariably associated with an active and acute state of the senses. In somnambulism all the senses are not commonly in full activity.

Summarily, then, these several states may be thus contrasted:—

In profound sleep there are no acts beyond excito-motor ones, and even these are reduced.

In somnambulism there are, beyond these, sensori-motor acts.

In dreaming ideas are aroused.

Dreaming, or a combination of the two, must be distinguished from purely somnambulistic acts. Perhaps in the most common form of somnambulism dreaming is, to a greater or less extent, combined with it. Thus somnambulism is presented under various forms, according to the absolute and relative degree of activity of the different senses and the condition of the cerebral lobes. All forms occur, from merely turning in sleep, to walking, talking, writing, and so forth.

And as in somnambulism some degree of activity of the cerebral lobes may be associated with an active state of the sensorium, so in dreaming some degree of activity of the sensorium may be combined with an active state of the cerebral lobes.

In those cases of so-called somnambulism in which acts are performed which involve a considerable exercise of the mental powers, the simple somnambulistic state must be combined with vivid dreaming. In this combination so many of the faculties are more or less active—so few, if any, completely at rest—that the individual is more awake than asleep.—*London Lancet.*

